# A Transfer Learning Approach with a Convolutional Neural Network for the Classification of Lung Carcinoma

**DOI:** 10.3390/healthcare10061058

**Published:** 2022-06-08

**Authors:** Mamoona Humayun, R. Sujatha, Saleh Naif Almuayqil, N. Z. Jhanjhi

**Affiliations:** 1 Department of Information Systems, College of Computer and Information Sciences, Jouf University, Sakakah 72312, Saudi Arabia; snmuayqil@ju.edu.sa; 2 School of Information Technology & Engineering, Vellore Institute of Technology, Vellore 632001, India; r.sujatha@vit.ac.in; 3 School of Computer Science (SCS), Taylor’s University, Subang Jaya 47500, Malaysia; noorzaman.jhanjhi@taylors.edu.my

**Keywords:** lung carcinoma, VGG 16, VGG 19, Xception, TL

## Abstract

Lung cancer is among the most hazardous types of cancer in humans. The correct diagnosis of pathogenic lung disease is critical for medication. Traditionally, determining the pathological form of lung cancer involves an expensive and time-consuming process investigation. Lung cancer is a leading cause of mortality worldwide, with lung tissue nodules being the most prevalent way for doctors to identify it. The proposed model is based on robust deep-learning-based lung cancer detection and recognition. This study uses a deep neural network as an extraction of features approach in a computer-aided diagnosing (CAD) system to assist in detecting lung illnesses at high definition. The proposed model is categorized into three phases: first, data augmentation is performed, classification is then performed using the pretrained CNN model, and lastly, localization is completed. The amount of obtained data in medical image assessment is occasionally inadequate to train the learning network. We train the classifier using a technique known as transfer learning (TL) to solve the issue introduced into the process. The proposed methodology offers a non-invasive diagnostic tool for use in the clinical assessment that is effective. The proposed model has a lower number of parameters that are much smaller compared to the state-of-the-art models. We also examined the desired dataset’s robustness depending on its size. The standard performance metrics are used to assess the effectiveness of the proposed architecture. In this dataset, all TL techniques perform well, and VGG 16, VGG 19, and Xception for 20 epoch structure are compared. Preprocessing functions as a wonderful bridge to build a dependable model and eventually helps to forecast future scenarios by including the interface at a faster phase for any model. At the 20th epoch, the accuracy of VGG 16, VGG 19, and Xception is 98.83 percent, 98.05 percent, and 97.4 percent.

## 1. Introduction

Cancer is a significant concern across the globe, and it is the second foremost reason for death. Lung cancer stands in third place among cancer deaths. Lung cancer is broadly classified into small and non-small-cell lung cancer. Various subtypes in non-small-cell lung cancer are adenocarcinoma, squamous cell cancer, and large cell carcinoma. Non-small-cell lung cancer is frequently observed, but small cell lung cancer spreads faster and is often fatal. Changes to the affected person’s voice, chest pain, shortness of breath, and wheezing are a few symptoms to list, and more painful symptoms also prevail [1,2]. Chemotherapy, targeted drugs, and immunotherapy are the treatments that have been approved over the decades [3].

**Figure 1:**
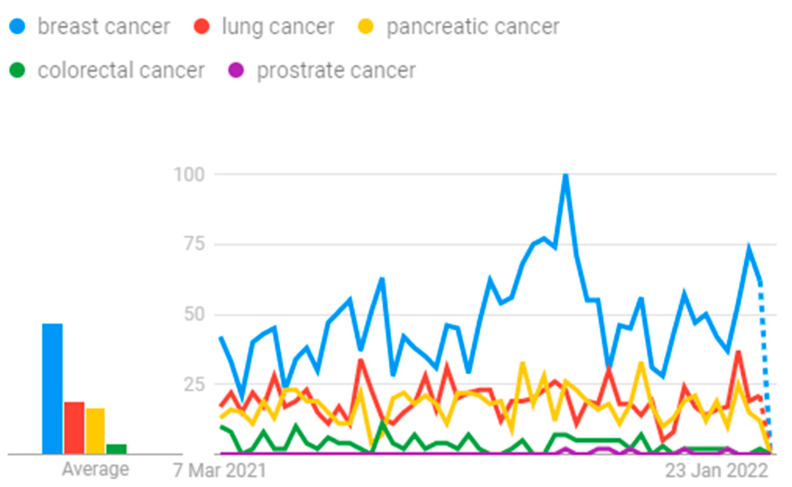
Cancer research.

Google Trends show the potential research going on in the field of cancer, and this is graphed in Figure 1. Several progressive methods are being devised for earlier detection with the help of artificial intelligence concepts.

Our main contribution to the work is as follows:

- We have designed a model which can classify the patients’ level of lung carcinoma by applying the TL process, and it is the first of the type to be carried out with this dataset [4].
- Using CT images as the system’s input, it can predict the level and helps to take contour action at the earliest time.
- We have applied three TL approaches here, namely VGG16, VGG19, and Xception, with 20 epochs in the Google Colab platform, and it was shown to be a better model to use for future prediction.
- Based on the experimental performance for lung carcinoma, VGG16 gives maximum accuracy of 98.83%, whereas Xception shows an accuracy rate of 97.4%.

The work is carried out in a phased manner. Section 2 of the paper discusses the recent work carried out in this domain. Section 3 details the workflow with architecture diagram illustration and fine-tuned hyperparameter values. Section 4 elaborates on the experimental discussion, followed by a comparison with recent results, conclusion, and future work in Section 5.

## 2. Related Work

Early diagnosis of the cancer cell is the required parameter irrespective of the organ. The initiation and growth of the tumor cell makes the life of the patient very difficult. Data filter algorithm for preprocessing, region growing algorithm for image segmentation, and feature selection followed by convolutional neural networks for classification are the various steps performed on computed tomography (CT) images to obtain proof of whether there is malignancy or not in the case of lungs [5]. Clinical attributes and the images of the work are used to predict the ailments at the earlier stage. Recent research in China has established a rapid blood test that screens lung cancer at the early stage. Smoking, radon exposure, lethal chemicals, particle pollution, and genetic factors are leading influences on lung cancer [4].

The EL-CAP dataset used in the work was created by Cornell University in 2003 and holds 500 low-dose images. The images were segregated in two folders based on the benign and malignant classes. For the work, 75% was considered as the training set and 25% as the testing set. A hybrid approach with a support vector machine and a feed-forward back propagation network was utilized to reduce the computational complexity in the classification process and produce accuracy of 98.08% [6]. The work was carried out with the enhanced spiral CT and MRI images of lung cancer obtained from 74 patients considered to be highly suspected of having lung cancer. CT and MRI accuracy was 94.6% and 89.2%, respectively. Data enhancement was performed. The implementation flow dumps data were followed by pretrained model loading and VGG-based TL accompanied by hyperparameter fine-tuning [7].

The risk factor associated with lung cancer was analyzed with the help of a database constructed from 1000 patients with 23 features related to the symptom and target as 3 values: low, medium, and high. Data visualization was performed, followed by decision trees and the random forest approach, to predict the lung cancer risk level. The accuracy obtained in this model was 93.33% [8]. The dataset considered was from sick people with non-small-cell lung cancer, who were treated with anti-programmed death therapy. Various attributes pertaining to the characteristics of patients, mutations, and results were retrieved from the laboratory. Out of several attributes, 19 were used to build the model, using machine learning algorithms such as ridge regression, multilayer neural network, LightBGM, XGBoost, linear discriminant analysis, and ridge regression. Feature engineering accompanied by machine learning shows that LightBGM serves well to build a highly reliable model for predicting lung cancer [9].

Artificial intelligence helps in diagnosis of the tumors at the earliest stage with the images collected from the cancer-prone area. Images captured via computed tomography, magnetic resonance, ultrasound, endoscopy procedure, and biopsies aid in better prediction. Breast, lung, thyroid, gastric, oral, skin, and liver cancer are interpreted with principles of artificial intelligence and expert inputs [10]. The deep transfer neural network and extreme learning machine were integrated and experimented on over the lung image database consortium and image database resource initiative dataset, and accuracy of 94.94% was achieved [11]. The cancer incidence data for this study were taken from the Surveillance, Epidemiology, and End Results (SEER) program. Statistics data are provided to combat the burden of cancer in the US at the early stages. Work carried out using ANN, RNN, and CNN paved the way for performance measures, and accuracy is 71.18% [12].

Several sources were used to compile a dataset of chest computed tomography scans with lung nodules and their pathologic diagnoses. We created, trained, and verified two machine learning algorithms. The support vector machine model was employed in the first algorithm, and the convolutional neural network was used in the second. The performance of the classification on the test dataset was evaluated using receiver operating characteristic analysis [13].

This work used the enhanced profuse clustering method (IPCT) and the deep learning with instantaneously trained neural networks (DITNN) strategy to evaluate lung CT images for predicting lung cancer. The lung CT pictures were first obtained from the Cancer Imaging Archive (CIA) collection and were then improved by constructing a weighted mean function that substituted the pixel using probability distribution and the cumulative distribution procedure. After improving the image’s representation, the damaged area was segregated using the pixel similarity value followed by the clusters that were established [14].

In order to provide prompt therapy to lung cancer patients, accurate detection of the disease is critical. Artificial neural networks (ANNs) are a recently developed machine learning algorithm that can be applied to both large and small datasets. An ensemble of weight optimized neural networks with maximum likelihood boosting for LCD in huge data is investigated in this research. Feature selection and ensemble classification are the two stages of the proposed technique. To reduce classification time, the important attributes were chosen in the first step using an integrated Newton–Raphson maximum likelihood and minimum redundancy (MLMR) preprocessing model. The patient was then classified using the boosted weighted optimized neural network ensemble classification algorithm in the second stage [15].

## 3. Workflow Architecture

**Figure 2:**
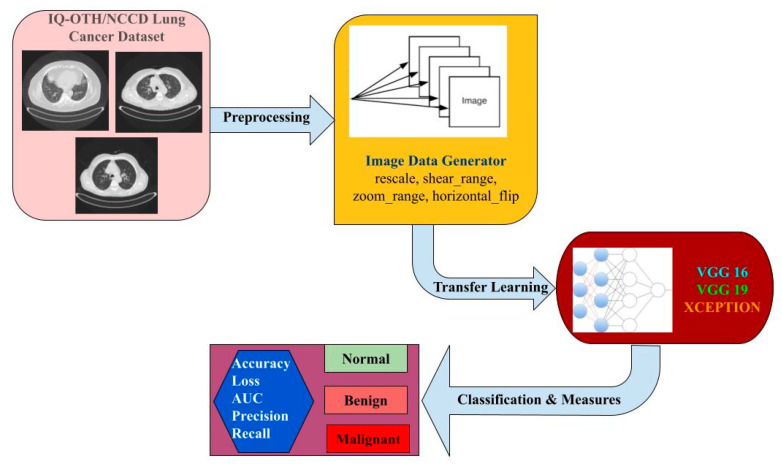
TL-based lung carcinoma classification.

Analysis of the cancer dataset with the TL was very informative and yielded a good model based on the various performance measure perspectives. Capturing the lung cancer image dataset was followed by preprocessing using the image data generator, then the TL model, and finally the prediction of the type of abnormality was achieved, as is portrayed in Figure 2.

### 3.1. Dataset Description

In the fall of 2019, the Iraq-Oncology Teaching Hospital/National Center for Cancer Diseases (IQ-OTH/NCCD) collected data for three months. The dataset comprised CT scans captured from patients in different stages and scans captured from patients in different stages and healthy people in DICOM format. The following CT protocol was utilized for reading: 120 kV, 1 mm slice thickness, window widths varying from 350 to 1200 HU, and window centers ranging from 50 to 600. An oncologist and radiologist working in the centers marked the slides. From 110 cases, 1190 images were retrieved. All the cases were classified into 3 classes, namely normal, benign, and malignant. In total, 55 cases were classified as normal, 15 cases were classified as benign, and 40 cases were classified as malignant.

Prior to analysis, all photos were de-identified. The oversight review board waived written consent. The institutional review boards of the participating medical centers approved the study. There were multiple slices in each scan. The number of slices varied between 80 and 200, and each one displayed an image of the human chest from various sides and perspectives. Gender, age, educational attainment, residency area, and living status were all different in the 110 cases. Some worked for the Iraqi ministries of transportation and oil, while others were farmers and gainers. Most of them were from Iraq’s central area, specifically the provinces of Baghdad, Wasit, Diyala, Salahuddin, and Babylon [16].

### 3.2. Image Data Generator

To expand the size of the dataset image, augmentation phase is deployed. In Keras, image data generator is used to achieve the augmentation. Different transformation is applied to the original to make it sizable in applying the deep learning structure. It also integrates variation in the images so that the built model will be reliable. This process requires reduced memory only. In our work, the training data following the augmentation technique have values as follows: rescale = 1.255, shear_range = 0.2, zoom_range = 0.2, and horizontal_flip = True. Upon analysis with various options, we found that this was most suitable. 

### 3.3. TL

To the created image dataset pool, TL algorithms, namely, VGG 16, VGG 19, and Xception were applied with fine-tuning hyperparameters to structure the network for training and testing in turn, in order to help in future prediction for new records. First, work was carried out with deep neural networks, meant for object detection. The alternative to deep learning is the TL method, and it works on four transfer methods, namely, instance-based, feature-based, parameter-based, and relation-based. Applications of TL are substantial and this article provides insight [17,18]. GPU support in the Google Colab platform provided the helping hand to overcome the computation glitches and showed good throughput.

### 3.4. Fine-Tuned Hyperparameters

Model hyperparameters were used to optimize the model, loss used was categorical_crossentropy, optimizer was adam. Fit_generator was the iterator method integrated to fit the data on the neural network simulation. Parameters were target_size = (224,224), batch_size = 32, and epochs = 20, and class_mode was categorical, based (normal, benign, malignant) on the nature of the dataset. In total, 75% of data were for training purposes. Varying hyperparameters were tried and executed to obtain optimized results.

The following performance measures were used to study the model consistency. The training and testing criteria showed varied performance [19,20].

#### 3.4.1. Accuracy

Accuracy is an ideal classification metric and is easy to understand [21,22]. It is the proportion of true results to total number of results. It is easily suited to binary and multiclass classification scenarios. It is a valid evaluation method for problems where data are not skewed and are well-balanced. Below is the confusion matrix to substantiate the 3-class performance measure.

(1) $$\mathrm{Accuracy}=\frac{TP+TN}{TP+FP+FN+TN}$$

ACTUAL VALUES

NormalBenignMalignantPREDICTED VALUESNormal+ve 1−ve 2−ve 3Benign−ve 4+ve 5−ve 6Malignant−ve 7−ve 8+ve 9

Normal

*TP* = Cell1
*FP* = Cell2 + Cell3
*TN* = Cell5 + Cell6 + Cell8 + Cell9
*FN* = Cell4 + Cell7

Benign

*TP* = Cell5
*FP* = Cell4 + Cell6
*TN* = Cell1 + Cell3 + Cell7 + Cell9
*FN* = Cell2 + Cell8

Malignant

*TP* = Cell9
*FP* = Cell7 + Cell8
*TN* = Cell1 + Cell2 + Cell4 + Cell5
*FN* = Cell3 + Cell6
*TP*—True Positive—predict yes and have lung carcinoma
*TN*—True Negative—correctly predict that they have no lung carcinoma
*FP*—False Positive—incorrectly predict having lung carcinoma but no lung carcinoma (type 1 error)
*FN*—False Negative—predict no lung carcinoma but have lung carcinoma (type 2 error)

#### 3.4.2. Loss

It is a function employed to compute distance between the current output of the algorithm and the expected output. It evaluates the performance of an algorithm’s ability to model data. There are two groups, one for classification and another for regression. The commonly used loss functions are cross-entropy, exponential loss, log loss, and hinge loss.

#### 3.4.3. AUC

It refers to area under the receiver operating characteristic curve. It is a performance indicator for how well the positive class probabilities are separated from negative class probabilities. It is scale-invariant. It indicates how well-ranked the predictions are, rather than the absolute values of predictions. Another benefit of employing it is that it is classification-threshold-invariant such that it measures the prediction’s quality irrespective of the threshold chosen.

#### 3.4.4. Precision

Precision is the ratio of relevant outcomes predicted to actually relevant outcomes [23,24]. It is also called positive prediction value. It takes false positives into account.

(2) $$\mathrm{Precision}=\frac{TP}{TP+FP}$$

#### 3.4.5. Recall

Recall gives the ratio of outcomes that were actually relevant to correctly predicted outcomes. It is also called sensitivity. It takes false negatives into account.

(3) $$\mathrm{Recall}=\frac{TP}{TP+FN}$$

#### 3.4.6. F1 Score

F1 Score is another measure that provides the harmonic mean of precision and recall.

(4) $$F1\;\mathrm{Score}=\frac{2*\mathrm{Precision}*\mathrm{Recall}}{\mathrm{Precision}+\mathrm{Recall}}$$

## 4. Experimental Discussion and Analysis

### 4.1. VGG 16

VGG 16 is a CNN model for visual recognition proposed by Karen Simonyan and Andrew Zisserman of Visual Geometry Group Lab at Oxford University in 2014. VGG 16 won first place in detecting objects within 200 classes and won second place in classifying images labelled 1 of 1000 categories at the ImageNet Large Scale Visual Recognition Challenge in 2014. Applying this model on the ImageNet dataset containing 1000 classes with 14 million images yielded 92.7% top-5 test accuracy.

It takes image input in dimensions (224,224,3). It has a total of sixteen layers. The first two layers are of 3∗3 filter size and have 64 channels with the same padding. Then there is the (2,2) stride max pool layer followed by two 256 filters and (3,3) filter size convolution layers. This is followed by a (2,2) stride max pool layer. Then there are two (3,3) size and 256 filters convolution layers. It is followed by two sets of three 512 filters and (3,3) size convolution layer and a max pool layer all of the same padding. The image is then passed to the two convolution layers stack. Padding of 1-pixel is used to prevent spatial features of the image after each convolution layer.

**Figure 3:**
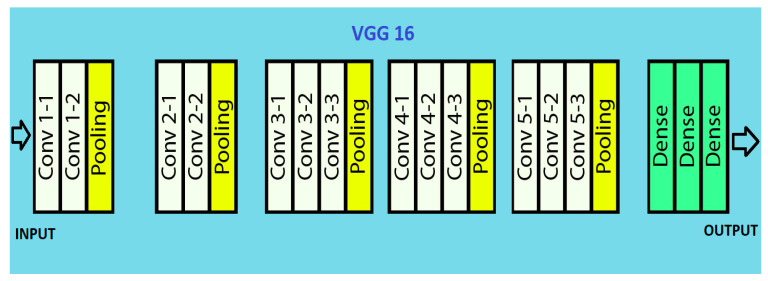
VGG 16 architecture.

The (7,7512) feature map is generated once the image passes through all the layers of VGG 16. This feature map is flattened to obtain the (1,25088) feature vector. There are three fully connected layers. The first obtains input from the last feature vector and gives a (1,4096) vector. The second also produces the same output as the first. While the third outputs 1000 channels for 1000 classes. The classification vector is then normalized, bypassing the output form third layer to softmax layer [25,26,27]. The architecture is pictured in Figure 3.

**Figure 4:**
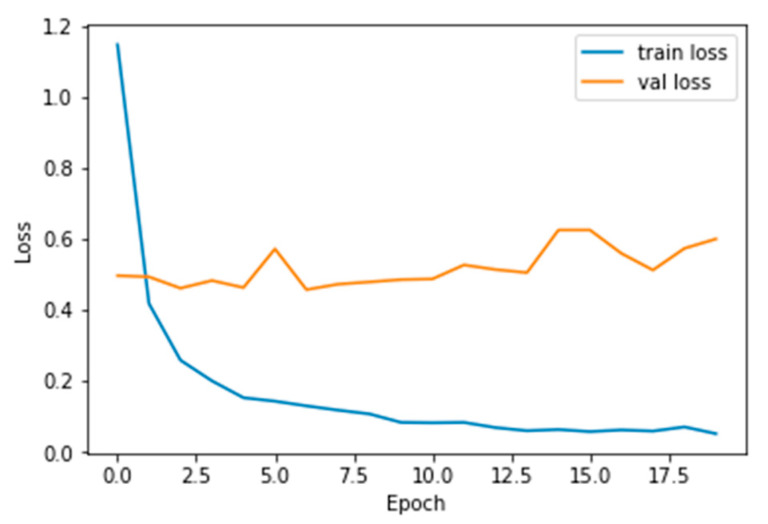
Loss vs. Epoch.

**Figure 5:**
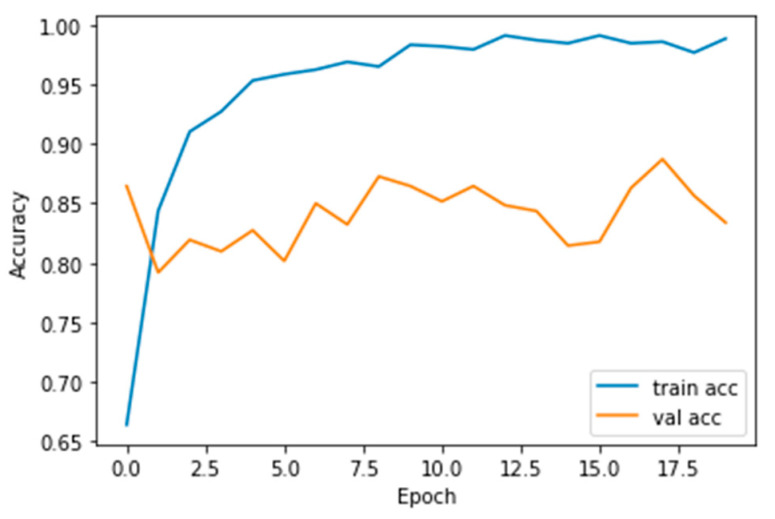
Accuracy vs. Epoch.

**Figure 6:**
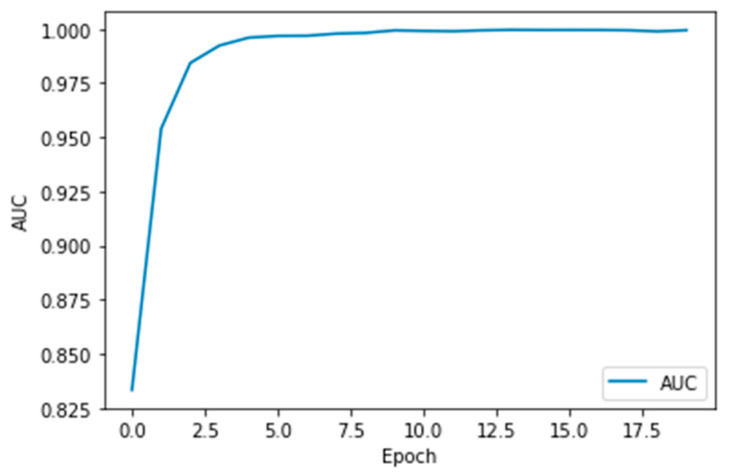
AUC vs. Epoch.

**Figure 7:**
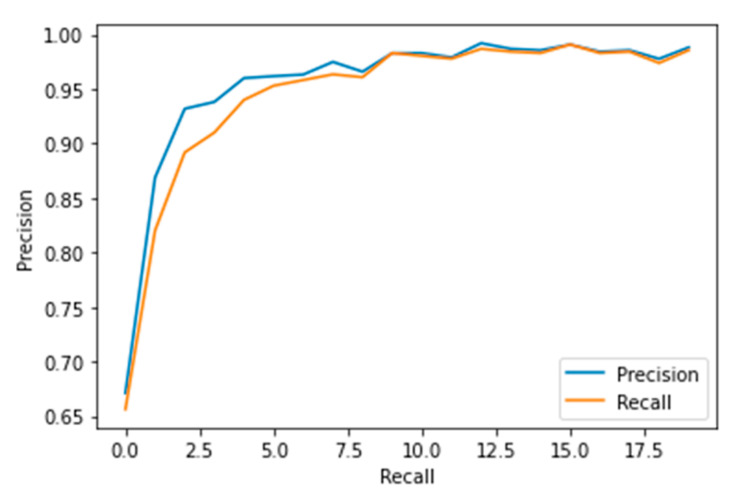
Precision vs. Recall.

The performance measures for the various epochs over VGG 16 are tabulated and plotted in Table 1 for training and Table 2 for testing data and are shown in Figure 4, Figure 5, Figure 6 and Figure 7. Gradually loss decreases, indicating the building of a good model; other performance measures also indicate the building of a good model and other performance measures show the increasing trend. By the start of the 5th epoch, the accuracy started to show outstanding value of above 95%. Similarly, AUC shows the ideal curve. Without compromising, precision and recall also show the upper trend.

### 4.2. VGG 19

A VGG model consisting of 19 layers is VGG 19. It has sixteen convolution layers, three fully connected layers, five maxpool layers, and a softmax layer. VGG 19 is conceptually the same as VGG 16 while the number of layers supported by it is different. Nineteen and sixteen denote the number of layers in the model. This model is trained on millions of images present in the ImageNet database. It can classify pictures into 1000 object categories such as pencil, keyboard, car, and many animals.

**Figure 8:**
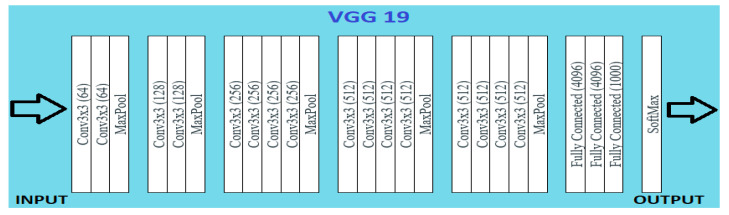
VGG 19 architecture.

The input RGB image is given in the fixed matrix dimension of (224,224,3). It is preprocessed by subtracting RGB mean value from each pixel. Kernels of (3,3) size with 1-pixel stride is used to cover the whole image notion. Spatial resolution of the image is preserved by the usage of spatial padding. The (2,2) max pooling is performed with two-pixel stride. This is followed by rectified linear unit (ReLu) to incorporate non-linearity in the model, which makes the model classify better and reduces computational time. This model proved to be much better than previously used models such as tanh or sigmoid function. It has three completely connected layers, of which the first two are of size 4096. A layer with 1000 channels for 1000 object categories of ILSVRC classification is also present, followed by a final softmax function, as illustrated in Figure 8.

Primarily, it was developed to win ILSVRC but later it was used as classification architecture for image datasets. Its models were made available to the public by the author, so it can be used as it is or with any required modifications for similar purposes. It can be used to perform TL tasks such as facial recognition. The VGG 19 model was able to perform facial recognition with higher accuracy even with mask usage [28].

**Figure 9:**
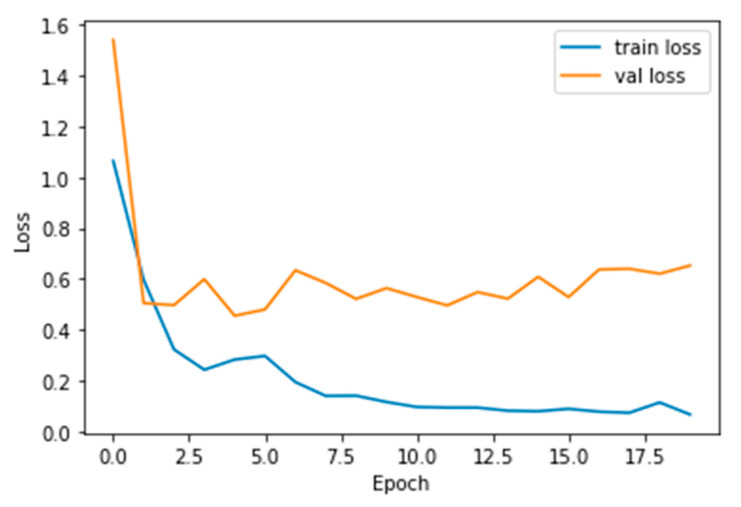
Loss vs. Epoch.

**Figure 10:**
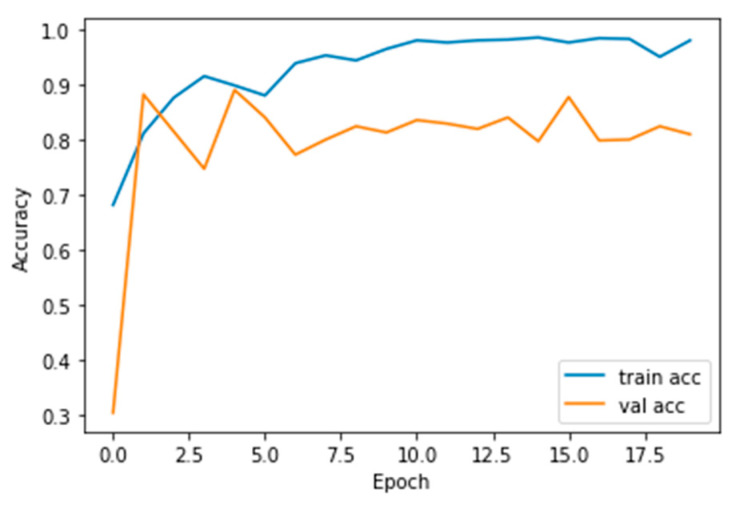
Accuracy vs. Epoch.

**Figure 11:**
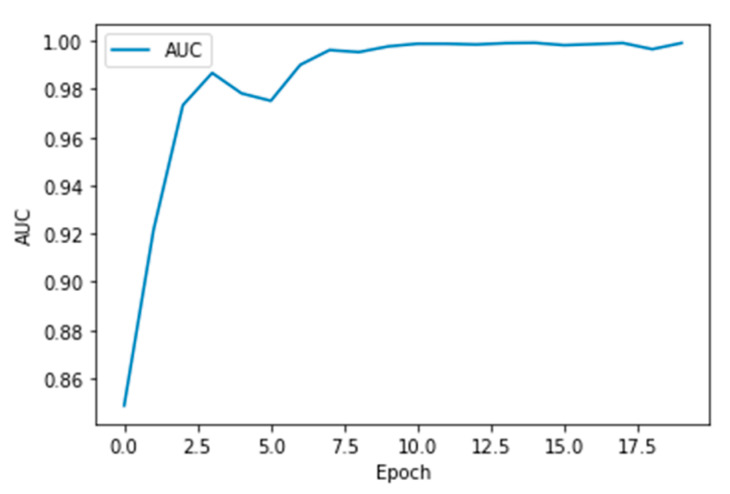
AUC vs. Epoch.

**Figure 12:**
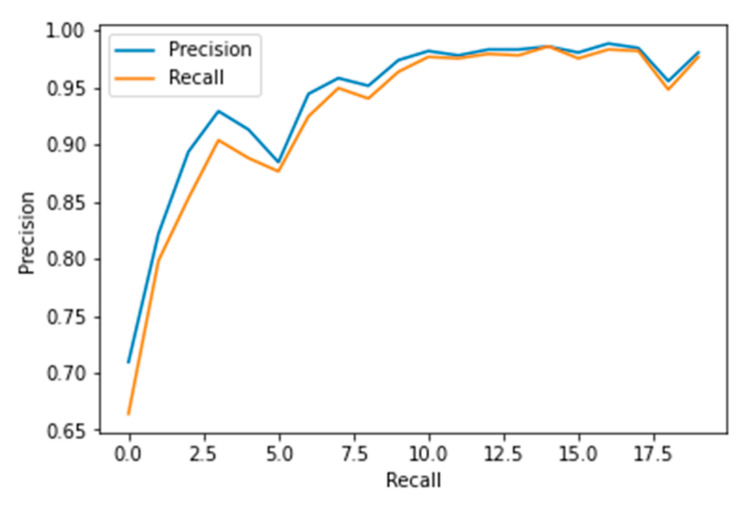
Precision vs. Recall.

Performance measures for the various epochs over VGG 19 are tabulated and plotted in Table 3 for training and Table 4 for testing data and are shown in Figure 9, Figure 10, Figure 11 and Figure 12. Gradually loss decreases, indicating the building of a good model; other performance measures also indicate the building of a good model and other performance measures show the increasing trend. By the start of the 10th epoch, the accuracy started to show outstanding value of above 95%. Similarly, AUC shows the ideal curve. Without compromising, precision and recall also show the upper trend.

### 4.3. Xception

It is a convolutional neural network model, with Google assisting object detection and image analysis. It is an extreme version of inception. It is based on layers of depth-wise separable convolution, which performs better than inception. It is also hypothesized that mapping of spatial correlations and cross-channel correlations in convolutional neural network feature maps can be completely decoupled. This hypothesis is stronger than the one present in inception.

Xception architecture possesses thirty-six convolution layers giving rise to a base where feature extraction can be performed. The thirty-six layers are separated into fourteen modules with linear residual connections surrounding them. Linear residual connections are exceptions in the first and last module. In short, there are depth-wise separable layers of convolution with residual connections in a linear stack. Hence, it is an easily definable and modifiable model. It can be modified by using just a few lines of code and high-level libraries such as Keras or TensorFlow.

**Figure 13:**
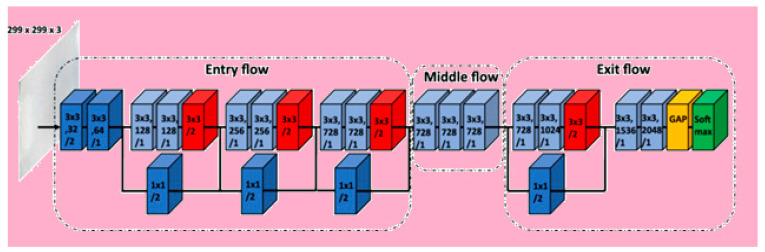
Xception architecture.

The presence of residual connections helps in convergence; it makes it a better performer in terms of classification and speed. The image first goes into entry flow, followed by middle flow, where the same process is repeated eight times. Finally, it goes through exit flow. All depth-wise separable convolution layers and convolution layers are followed by batch normalization. All layers have a depth multiplier of one, such that there is no expansion of depth. Xception models perform well in convolutional neural networks, and Figure 13 depicts the flow [29].

**Figure 14:**
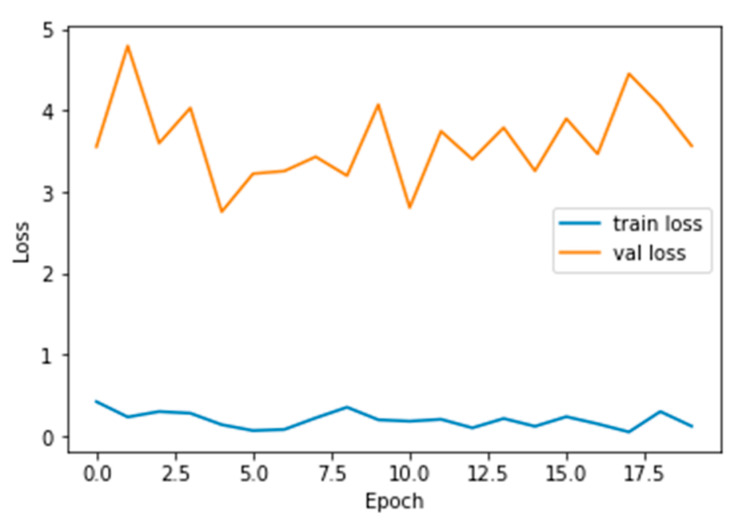
Loss vs. Epoch.

**Figure 15:**
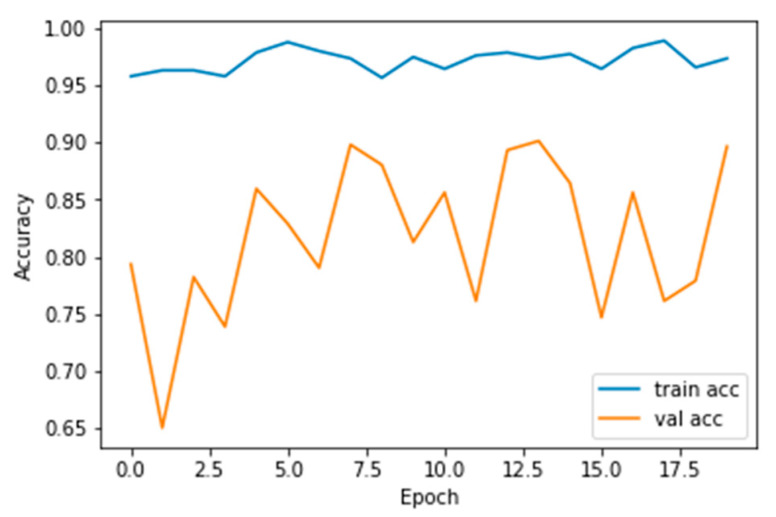
Accuracy vs. Epoch.

**Figure 16:**
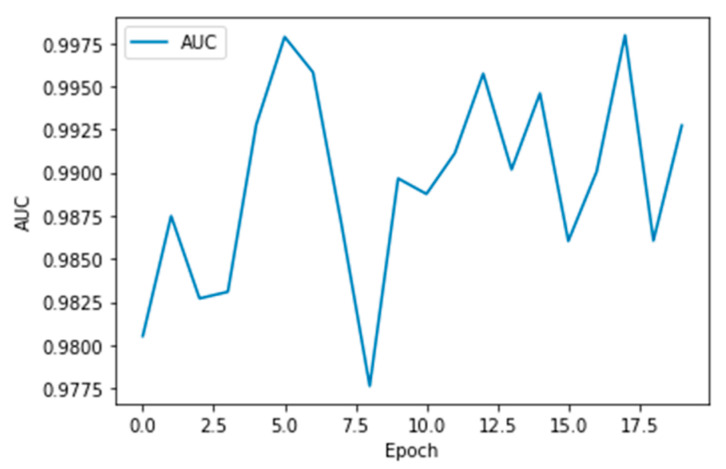
AUC vs. Epoch.

**Figure 17:**
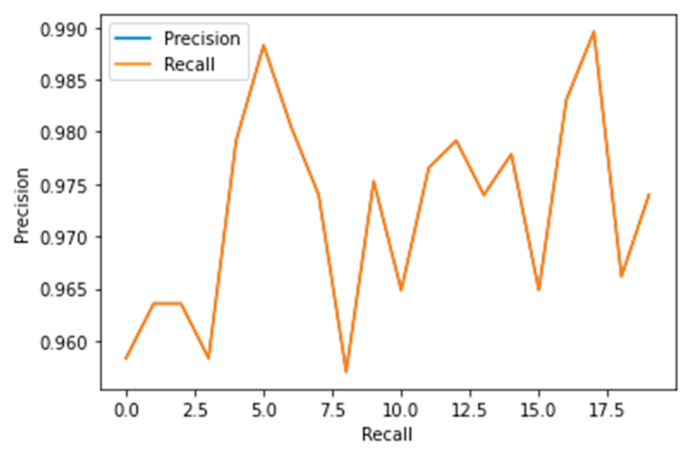
Precision vs. Recall.

The performance measures for the various epochs over Xception are tabulated and plotted in Table 5 for training and Table 6 for testing data and are shown in Figure 14, Figure 15, Figure 16 and Figure 17. Gradually loss decreases, indicating the building of a good model; other performance measures also indicate the building of a good model and other performance measures show the increasing trend. From the beginning itself, accuracy, AUC, precision, and recall are good, and as mentioned, this model performs well in the initial stage itself. Because of the similar values, the Figure 17 graph merges precision vs. recall together.

## 5. Conclusions and Future Work

On comparison with the recent works carried out using this same dataset, our model yields good results. With the same dataset work carried out using SVM classification, the accuracy achieved is 89.8876% [30]. In work carried out with the AlexNet architecture, accuracy is 93.548% [31].

All TL approaches perform well in this dataset and compare VGG 16, VGG 19, and Xception for the 20-epoch structure. For any model, preprocessing acts as the great bridge to construct a reliable model and eventually helps predict a reliable model and eventually helps in predicting future cases by incorporating the interface at the faster phase. Cumulatively, accuracy of VGG 16, VGG 19, and Xception is 98.83%, 98.05%, and 97.4%, respectively, at the 20th epoch. Loss is somewhat high in the case of Xception over testing data, and accuracy of VGG 16, VGG 19, and Xception is 83.39%, 80.97%, and 89.68%, respectively, at the 20th epoch. In comparison, VGG16 and VGG19 outperform.

Utilization of other clinical data along with this will provide even more clarity. Additional demographic information helps in taking additional steps to create awareness in more prone locations. Normally, lifestyle factors such as eating habits, cultural habits, and social living have a great impact on many diseases and cumulating the same will aid greatly in decision-making.

## Figures and Tables

**Table 1:** Performance measures over VGG 16 for training data.

Epochs	Loss	Accuracy	AUC	Precision	Recall	F1 Score
1	1.1475	0.6641	0.8333	0.6711	0.6562	0.6636
5	0.1522	0.9531	0.996	0.9601	0.9401	0.9500
10	0.0826	0.9831	0.9993	0.9831	0.9831	0.9831
15	0.0625	0.9844	0.9995	0.9856	0.9831	0.9843
20	0.0508	0.9883	0.9994	0.9883	0.9857	0.9870

**Table 2:** Performance measures over VGG 16 for testing data.

Epochs	Loss	Accuracy	AUC	Precision	Recall	F1 Score
1	0.4963	0.8645	0.9409	0.882	0.8435	0.8623
5	0.4626	0.8274	0.9454	0.8394	0.8177	0.8284
10	0.4851	0.8645	0.9472	0.8695	0.8597	0.8646
15	0.6248	0.8145	0.923	0.8139	0.8113	0.8126
20	0.5997	0.8339	0.939	0.8363	0.8323	0.8343

**Table 3:** Performance measures over VGG 19 for training data.

Epochs	Loss	Accuracy	AUC	Precision	Recall	F1 Score
1	1.0643	0.681	0.8486	0.7093	0.6641	0.6860
5	0.2821	0.8984	0.9783	0.913	0.888	0.9003
10	0.115	0.9648	0.9978	0.9737	0.9635	0.9686
15	0.0785	0.9857	0.9992	0.9857	0.9857	0.9857
20	0.0658	0.9805	0.9992	0.9804	0.9766	0.9785

**Table 4:** Performance measures over VGG 19 for testing data.

Epochs	Loss	Accuracy	AUC	Precision	Recall	F1 Score
1	1.5413	0.3032	0.5908	0.289	0.2661	0.2771
5	0.4545	0.8903	0.9494	0.8988	0.8742	0.8863
10	0.5633	0.8129	0.9296	0.823	0.8097	0.8163
15	0.6081	0.7968	0.9319	0.798	0.7839	0.7909
20	0.6524	0.8097	0.9262	0.811	0.8097	0.8103

**Table 5:** Performance measures over Xception for training data.

Epochs	Loss	Accuracy	AUC	Precision	Recall	F1 Score
1	0.4247	0.9583	0.9805	0.9583	0.9583	0.9583
5	0.1418	0.9792	0.9928	0.9792	0.9792	0.9792
10	0.2022	0.9753	0.9897	0.9753	0.9753	0.9753
15	0.1218	0.9779	0.9946	0.9779	0.9779	0.9779
20	0.1238	0.974	0.9927	0.974	0.974	0.974

**Table 6:** Performance measures over Xception for training data.

Epochs	Loss	Accuracy	AUC	Precision	Recall	F1 Score
1	3.5571	0.7935	0.8709	0.7935	0.7935	0.7935
5	2.7582	0.8597	0.9236	0.8597	0.8597	0.8597
10	4.0757	0.8129	0.8893	0.8129	0.8129	0.8129
15	3.2605	0.8645	0.9207	0.8643	0.8643	0.8643
20	3.5682	0.8968	0.9338	0.8968	0.8968	0.8968

## Data Availability

Data will be furnished on request.
